# Involvement of the Nonneuronal Cholinergic System in Bone Remodeling in Rat Midpalatal Suture after Rapid Maxillary Expansion

**DOI:** 10.1155/2016/8106067

**Published:** 2016-07-10

**Authors:** Xiaoxia Che, Jie Guo, Lue Wang, Cong Miao, Lihua Ge, Zhenchuan Tian, Jianhong Wang

**Affiliations:** ^1^Department of Orthodontics, School of Stomatology, Capital Medical University, Beijing 100050, China; ^2^Department of Orthodontics, School of Stomatology, Shandong University, Shandong Provincial Key Laboratory of Oral Biomedicine, Jinan 250012, China; ^3^College of Life Science and Technology, Beijing University of Chemical Technology, Beijing 100029, China; ^4^Institute of Dental Research, Beijing Stomatological Hospital and School of Stomatology, Capital Medical University, Beijing 100050, China

## Abstract

Few studies sought to analyze the expression and function of the nonneuronal acetylcholine system in bone remodeling* in vivo *due to the lack of suitable models. We established a rat maxilla expansion model in which the midline palatine suture of the rat was rapidly expanded under mechanical force application, inducing tissue remodeling and new bone formation, which could be a suitable model to investigate the role of the nonneuronal acetylcholine system in bone remodeling* in vivo. *During the expansion, the expression pattern changes of the nonneuronal cholinergic system components and the mRNA levels of OPG/RANKL were detected by immunohistochemistry or real-time PCR. The value of the RANKL/OPG ratio significantly increased after 1 day of expansion, indicating dominant bone resorption induced by the mechanical stimulation; however after 3 days of expansion, the value of the RANKL/OPG ratio significantly decreased, suggesting a dominant role of the subsequent bone formation process. Increasing expression of Ach was detected after 3 days of expansion which indicated that ACh might play a role in bone formation. The mRNA expression levels of other components also showed observable changes during the expansion which confirmed the involvement of the nonneuronal cholinergic system in the process of bone remodeling* in vivo*. Further researches are still needed to figure out the detailed functions of the nonneuronal cholinergic system and its components.

## 1. Introduction

Traditionally acetylcholine (ACh) is regarded as a classical neurotransmitter in the central nervous system, the postganglionic parasympathetic system, and the preganglionic neurons of the sympathetic system, in sweat glands of the human axilla, and in several areas of the brain, mediating chemical neurotransmission at neurons, ganglia, interneurons, and the motor endplate via nicotinic ACh receptors (nAChRs) or mediating chemical neurotransmission at neurons and effector organs via muscarinic ACh receptors (mAChRs). However, the sole role of ACh as a neurotransmitter has been challenged since the last century based on the discoveries that all components of the cholinergic system (including ACh-synthesizing enzymes, transporters, receptors and degrading enzymes) were demonstrated in mammalian nonneuronal cells and this system has been defined as the nonneural cholinergic system [1]. ACh can act as an auto/paracrine mediator in nonneuronal cells such as embryonic stem cells [2], epithelial cells [3], endothelial cells [4], immune cells [5], mesothelial cells [6], and mesenchymal cells [7]. Recent researches found that ACh synthesized by nonneuronal cells could modulate many important biological and physiological processes such as cell growth, adhesion, migration, and differentiation in response to the internal or external stimuli via different ACh receptors [1]. Moreover, the malfunctions of the nonneuronal cholinergic system are involved in the pathogenesis and pathology of various diseases such as atopic dermatitis [8], psoriasis [9], inflammatory bowel diseases [10], colon cancer [11], and cardiac diseases [12].

Previous studies also offer some clues to the role of the nonneuronal cholinergic system in the bone metabolism. Inkson et al. identified the expression, secretion, and adhesive function of AChE in osteoblasts, proposing the regulation role of AChE in cell-matrix interaction in the bone [13]. En-Nosse et al. found that all necessary enzymes, transporters, and receptors for ACh synthesis and recycling could be detected in osteoblast-like cells [14]. Expressions of ACh receptors nAChRs and mAChRs were also confirmed in primary bone cells, mesenchymal stem cells, and osteoclasts [15]. The extensive expressions of cholinergic components in bone tissue suggest the functional role they may play in bone metabolism [1]. Besides, previous study revealed that ACh regulated the migration of bone marrow-derived mesenchymal stem cells which could differentiate into bone cells [16]. Nicotinic stimulation in mice could induce bone mass accrual which might be attributed to increasing osteoclast apoptosis [17], whereas* in vitro* muscarinic stimulation promoted osteoblast proliferation [18]. These studies strongly indicated an important role nonneuronal cholinergic system could play in bone metabolism and bone remodeling.

However, most of these studies were conducted* in vitro*; few researches sought to analyze the expression and function of the nonneuronal acetylcholine system in bone remodeling* in vivo *due to the lack of suitable models. Our previous work demonstrated the extensive expression of cholinergic system components in rat maxilla* in vivo* [19]. Based on the previous results, a rat maxilla expansion model was employed in which the midline palatine suture of the rat was rapidly expanded under mechanical force application, inducing tissue remodeling and new bone formation in the midpalatal suture [20], to investigate the role of nonneuronal cholinergic system in bone remodeling* in vivo* by detection of the expression pattern changes of the nonneural cholinergic system components in the maxilla during the expansion.

## 2. Materials and Methods

### 2.1. Experimental Animals and Group

Thirty-six 8-week-old male Sprague-Dawley rats purchased from Vital River Laboratory (Beijing, China) with a mean weight of 208.36 ± 7.32 g were divided into 4 experimental groups of 9 animals each as follows: C, control group without treatment; E1, group in which the rats underwent midpalatal expansion for 1 day; E3, group in which the rats underwent midpalatal expansion for 3 days; E7, group in which the rats underwent midpalatal expansion for 7 days. The animal protocol was in accordance with the guideline of animal ethics defined by the Ethics Committee of Capital Medical University. In each group 3 specimens were used for immunohistochemistry and the other 6 were used for quantitative real-time reverse transcription PCR.

### 2.2. Midpalatal Suture Expansion

The rats were subjected to midpalatal sutural expansion as described in the previous study [21]. The distal ends of the midpalatal expansion appliance (0.45 mm stainless steel A. J. Wilcock Australian Wire, A. J. Wilcock PTY, Ltd., Melbourne, Australia) were placed into the interproximate space between the second and third molars and then activated through the ends of the compression helices to exert an initial expansion force of 150 g. A 1.5 cm midsagittal incision was made anteroposteriorly after appliance placement to eliminate the side effects such as biaxially tipping of anchored teeth following midpalatal expansion and to make the expansion force applied to the maxilla as possible as it could. Expansion appliances were activated twice every other day.

### 2.3. Immunohistochemistry

Dissected maxilla samples including the midpalatal suture from the first to second molars of rats from all groups were fixed in 10% formalin solution for 48 hours. Routine paraffin embedding procedures were conducted. Sections (5 *µ*m thick) were cut and mounted on poly-L-lysine-coated glass slides. The prepared paraffin sections were tested for Ach immunohistochemistry. Sections were dewaxed with xylene and rehydrated sequential immersion in gradient ethanol. The endogenous peroxidase activity was blocked by incubation in a 3% hydrogen peroxide buffer for 10 minutes and then incubated with ACh antibodies (Chemicon) in a humidified chamber at 4°C overnight. Sections were rinsed with phosphate buffer solution (PBS) 3 times and then incubated in dark for 1 h at 37°C with secondary antibodies (Maixin Biotechnology Inc., Fuzhou, China). Sections were counterstained with hematoxylin, dehydrated, cleared, and mounted.

To better describe the expression pattern of ACh in the maxilla, the midpalatal shelf was divided into 4 different areas (Figure 2(a)) in each section when analysis was conducted. Area 1 represented the cartilage of the midpalatal suture under the nasal mucosa. The bone tissues beside the suture at the oral mucosa side were marked as Area 2 and as Area 3 at the nasal mucosa side between which was the bone marrow-like cavities containing abundant mononuclear cells. Area 4 corresponded to the maxilla compact bones above the palatine vessels and nerve bundles. Images were collected with a OLYMPUS BX61 optical microscope (Tokyo, Japan) and analyzed using Image Pro Plus 5.0 software (Media Cybernetics, Inc., Silver Spring, MD). The intensity of staining was determined by counting the mean optical density (MOD) in 5 different fields for each section. Batch processing was applied by macroprocessor Pathology 6 and MOD values were obtained for each visual field at a magnification of 100x and the mean value of MOD was calculated.

### 2.4. RNA Extraction, Reverse Transcription, and Real-Time PCR

Dissected maxilla samples were harvested in each group, and soft tissues covering the maxillae were stripped off with a blade to eliminate contamination. Specimens were ground under liquid nitrogen, and total RNAs were extracted with Trizol reagent (Invitrogen, Carlsbad, CA, USA). RNAs were reversely transcribed to cDNA with SuperScript reverse transcriptase III (Invitrogen, Carlsbad, CA, USA) according to the manufacturer's instructions. The cDNAs were amplified with gene-specific primers (Table S1 in Supplementary Material available online at http://dx.doi.org/10.1155/2016/8106067). Reactions were run on the Roche 480 real-time PCR detection system (ROCHE GROUP, Basel, Switzerland). The running conditions were as follows: 50°C for 2 minutes and 95°C for 5 minutes, followed by 45 cycles of 95°C for 15 s and 60°C for 1 minute. Rat *β*-actin gene and 18S gene were used as internal controls.

### 2.5. Statistical Analysis

Data were analyzed with SPSS 17.0 software. The normal distribution of the data was analyzed by Shapiro-Wilk Test. For data accorded with normal distribution, the statistical significance between the experimental groups was analyzed with one-way ANOVA test. For data not accorded with normal distribution the statistical significance between the experimental groups was analyzed with Kruskal-Wallis Test. Differences with *P* values less than 0.05 were considered as significant.

## 3. Results

### 3.1. Bone Remodeling Pattern in Rat Maxilla after Rapid Maxillary Expansion

**Figure 1:**
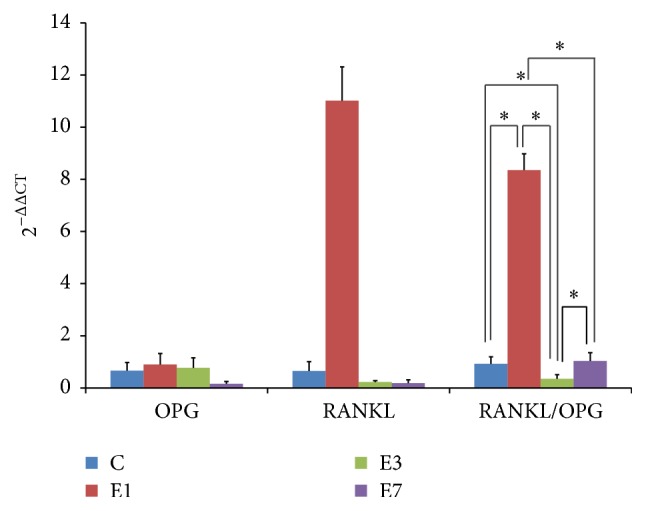
The change pattern of RANKL/OPG in the rat maxillae during the maxillary expansion. C: control group without treatment. E1: group in which the rats underwent midpalatal expansion for 1 day. E3: group in which the rats underwent midpalatal expansion for 3 days. E7: group in which the rats underwent midpalatal expansion for 7 days.  ^*∗*^Statistically significant.

To evaluate the bone remodeling pattern and the spatial and temporal change of balanced bone resorption and formation process in the midpalatal expansion, qRT-PCR was performed to determine the ratio of RANKL and OPG expression. Results (Figure 1) showed that value of the RANKL/OPG ratio significantly increased in the rat maxilla after 1-day expansion which indicated obviously bone resorption induced by the mechanical stimulation. After 3-day expansion, the value of the RANKL/OPG ratio significantly decreased, suggesting a dominant role of the subsequent bone formation process. The value of the RANKL/OPG ratio returned to the initial base line after 7-day midpalatal expansion.

### 3.2. ACh Expression Pattern in Rat Maxilla

**Figure 2:**
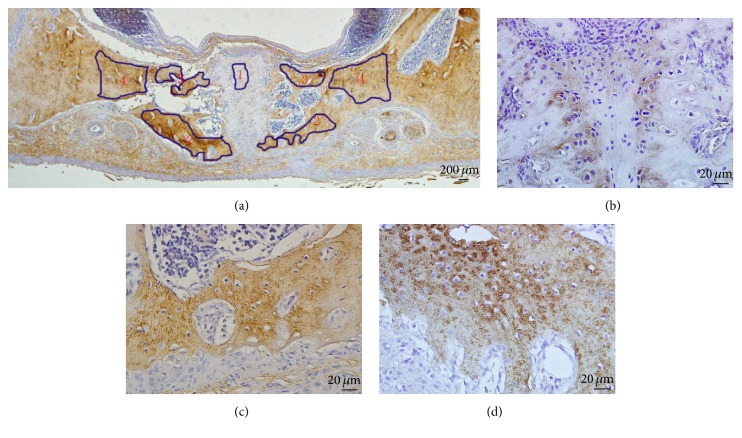
Ach expression in the rat maxilla. (a) Area partition of midpalatal shelf after immunohistochemical staining; (b) ACh expression in the cytoplasm of chondrocytes and the cartilage matrix in midpalatal suture; (c) the vesicle release pattern of ACh recognized as the browny round rots in the bone tissues; (d) the vesicle release pattern of ACh in compact maxilla beside the alveolar bone.

The morphological structure of rat maxilla was divided into 4 areas and shown in Figure 2(a). Area 1 represented the cartilage of the midpalatal suture under the nasal mucosa; Area 2 represented the bone tissues beside the suture at the oral mucosa side; Area 3 represented the bone tissues beside the suture at the nasal mucosa side; Area 4 corresponded to the maxilla compact bones above the palatine vessels and nerve bundles. Abundant ACh expression could be observed in the cytoplasm of chondrocytes and the cartilage matrix in midpalatal suture in Area 1 in the control group (Figure 2(b)). The vesicle release pattern of ACh could be detected as the browny round rots shown in the bone tissues in Area 2 after 1-day expansion (Figure 2(c)) and in the compact maxilla beside the alveolar bone in Area 4 after 1-day expansion (Figure 2(d)).

### 3.3. Changes of Ach Expression Level during the Rat Midpalatal Suture Expansion

**Figure 3:**
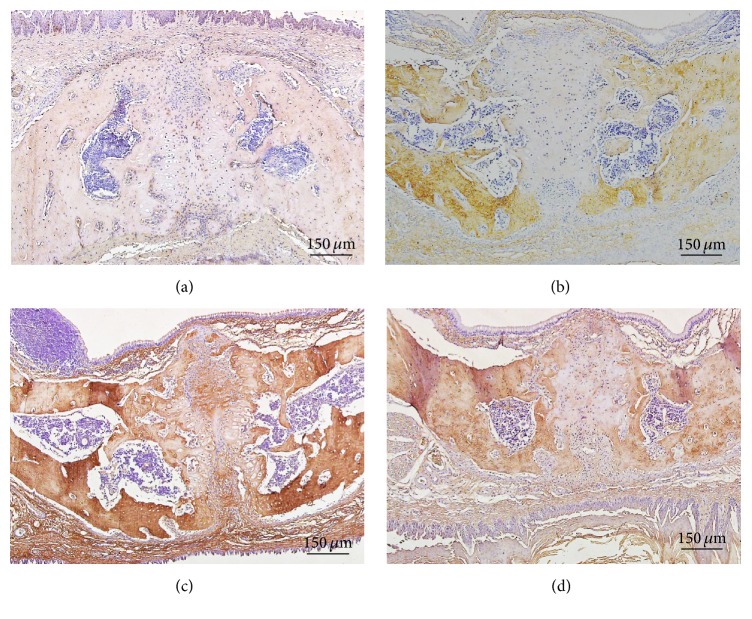
Expression changes of Ach during the expansion of rat midpalatal suture. (a) ACh expression in the rat maxilla in control group; (b) enhanced ACh expression in midpalatal suture after 1 d expansion; (c) ACh expression in midpalatal suture after 3 d expansion; (d) ACh expression in midpalatal suture after 7 d expansion.

**Figure 4:**
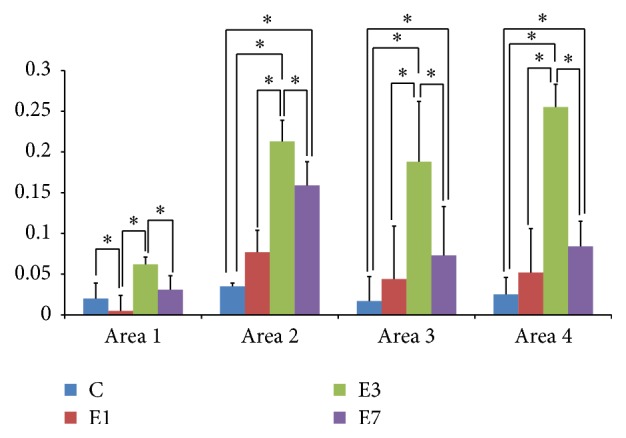
The quantity assessment of ACh expression during the expansion process with Image-pro plus software. C: control group without treatment. E1: group in which the rats underwent midpalatal expansion for 1 day. E3: group in which the rats underwent midpalatal expansion for 3 days. E7: group in which the rats underwent midpalatal expansion for 7 days.   ^*∗*^Statistically significant.

Compared with control group (Figure 3(a)), increased ACh expression could be detected in the maxillary suture and bone tissues after 1 d expansion (Figure 3(b)), 3 d expansion (Figure 3(c)), and 7 d expansion (Figure 3(d)). The quantitative analysis results (Figure 4) showed that after 1 day of expansion there was a significant increase of ACh expression in bone tissue in Areas 3 and 4. Ach expression in midpalatal bone tissue in all the 4 areas was statistically strengthened after 3 days of expansion. However, ACh expression in the bone tissues in Areas 2, 3, and 4 decreased significantly after 7 days of expansion compared with the results after 3 days of expansion, which was still higher than in the control group.

### 3.4. Expression Changes of Other Cholinergic Components during the Rat Midpalatal Suture Expansion

**Figure 5:**
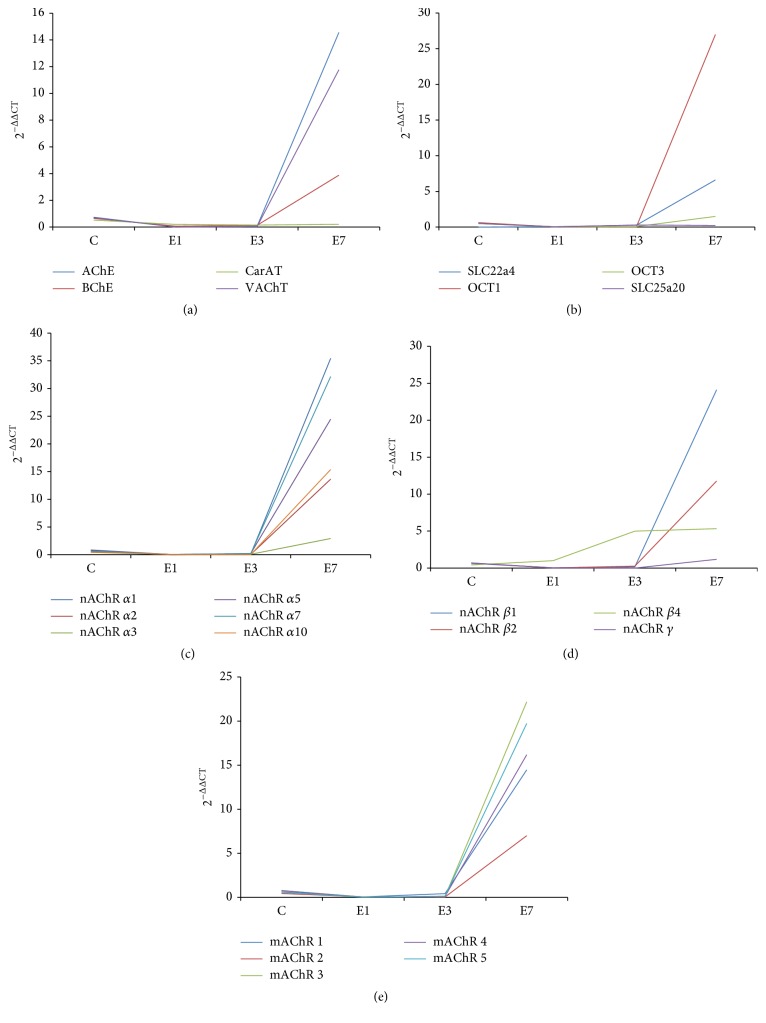
Expression changes of other cholinergic components in the expansion of rat midpalatal suture detected by RT-PCR. ((a) and (b)) Expression of ACh synthesis, transport and degradation systems in the rat maxillae during the maxillary expansion, including synthesis component CarAT, transport components VAChT, OCT1, OCT3, SLC22a4, and SLC25a20, and degradation components AchE and BchE. ((c) and (d)) Expression of nicotinic acetylcholine receptors in the rat maxillae during the maxillary expansion. (e) Expression of muscarinic acetylcholine receptors in the rat maxillae during the maxillary expansion. C: control group without treatment. E1: group in which the rats underwent midpalatal expansion for 1 day. E3: group in which the rats underwent midpalatal expansion for 3 days. E7: group in which the rats underwent midpalatal expansion for 7 day.

The expression levels of 34 genes of the nonneuronal cholinergic system components, including ChAT, carnitine acetyltransferase (CarAT), AChE, CHT, organic cation transporters (OCT), and n- and mAChRs, in the rat maxilla during the expansion were detected with real-time quantitative PCR. RNA expressions of CHT, ChAT, OCT2, SLC25a29, and nAChR subunits *α*4, *α*6, *α*9, *β*3, *δ*, and *ε* were detected in rat maxilla. Abundant expressions of CarAT, VAChT, AChE, OCT1, and nAChR subunits *α*1, *α*2, *α*5, *α*7, *α*10, *β*1, *β*2, and *γ* and mAChR subunits M2, M3, M4, and M5 were observed. The changing tendency of these genes induced by midpalatal expansion was showed in Figure 5. AChE, BChE, VAChT (Figure 5(a)), SLC22a4 (Octn1), and OCT1 (Figure 5(b)) gene expression decreased significantly after 1 day of midpalatal expansion and then went up slightly by 3 days of expansion, but still lower than in the control group. After 7 days of expansion, expression of these genes increased obviously. The same change pattern could be detected in the expression of nAChR subunits *α*1, *α*2, *α*5, *α*7, *α*10 (Figure 5(c)), *β*1, and *β*2 (Figure 5(d)) and mAChR subunits M2, M3, M4, and M5 (Figure 5(e)). Expression of CarAT (Figure 5(a)) and SLC25a20 (Figure 5(b)) showed no significant change before and after the midpalatal expansion though abundant expression of these genes was detected. nAChR subunits *γ* (Figure 5(d)) showed significant increase after 7 days of midpalatal expansion.

## 4. Discussion

Although several studies have demonstrated the presence of all the nonneuronal system components in osteoblasts and bone tissues, few pieces of information could be discovered to explain the potential role acetylcholine may play in bone metabolism and bone remodeling. Besides, most of these studies were conducted* in vitro*; few researches sought to analyze the expression and function of the nonneuronal acetylcholine system in bone remodeling* in vivo *which may bedue to the lack of suitable models. In this study, we employed a rat maxilla expansion model in which the midline palatine suture of the rat was rapidly expanded under mechanical force application, inducing tissue remodeling and new bone formation in the midpalatal suture [20] to investigate the role of the nonneuronal cholinergic system in bone remodeling. The value of RANKL/OPG ratio based on the mRNA expression levels was calculated to indicate the spatial and temporal change of balanced bone resorption and formation process in the expansion process. The OPG/RANK/RANKL signaling pathway is a key regulator of osteoblast and osteoclast activity in the normal bone turnover [22]. Elevated RANKL/OPG ratio has been found to indicate predominant bone resorption over bone formation during bone remodeling [23]. In this study, obvious increase of the value of RANK/OPG ratio was observed, suggesting a dominant role of bone resorption after 1 day of midpalatal expansion. After 3 days of expansion, the value of RANKL/OPG ratio significantly decreased, suggesting a dominant role of the subsequent bone formation process. After 7 days of midpalatal expansion, RANKL/OPG levels returned to the initial levels, indicating that the bone formative activities were renewed.

Extensive expression of ACh was observed in rat maxilla, mainly in the cytoplasm of chondrocytes, the cartilage matrix, and midpalatal bone matrix. ACh expression increased obviously after the expansion with a vesicle release pattern and reached a peak after 3 days of expansion, indicating that ACh might play an important role in the process of bone formation. A previous study [24] has proposed that acetylcholine could cause the reciprocal regulation of RANKL and OPG mRNA expression, resulting in a significant increase in the value of RANKL/OPG value in osteocytes via acetylcholine receptors.

ACh is efficiently synthesized by ChAT in the nervous system. Sato et al. reported that ChAT mRNA could be detected in both immature and mature osteoblasts [18]; however in our work ChAT mRNA was not detected in chondrocytes in the midpalatal suture and osteoblasts. Alternatively, carnitine acetyltransferase (CarAT) which provided the alternative route for ACh synthesis was detected in the midpalatal bone tissue. It was reported that CarAT could also be detected in skeletal muscle fibres [25], urothelium [26], and human (SAOS-2) and murine (MC3T3-E1) osteoblast-like cells [14]. Though abundant expression of CarAT could be detected in the midpalatal suture, no obvious change was observed during the midpalatal expansion.

After being synthesized from choline and acetyl CoA by ChAT or CarAT in the cytoplasm, acetylcholine can be transported to presynaptic vesicles by VAChT and released into the synaptic cleft or directly released by OCTs. In our work, abundant mRNA expression of VAChT was detected and the transport vesicle of ACh could be observed by immunohistochemistry, indicating that ACh was stored and released by VAChT in the maxilla.

Both AChE and BChE which undertook acetylcholine degradation were detected in the rat midpalatal bone tissues. High-affinity choline transporter (CHT) is responsible for the recycle of choline derived from ACh hydrolysis in the extracellular cleft to maintain continued production and release of Ach [27]. However, CHT expression was not detected in the midpalatal bone tissue. Choline-specific transporter-like proteins (CTL family) and polyspecific organic cation transporters (OCT family) are the other two families of choline transporters beside CHT [28]. In our work, OCT-1 and SLC22a4 (Octn1) were detected in rat maxilla, suggesting that uptaking of choline from extracellular cleft in rat maxilla might be performed by OCT family. Beckmann et al. reported that, in the human joint, the transport of choline into the cell and the release of ACh seem to be mediated mainly by OCT1, OCT3, and SLC22a4 (Octn1) and all members of the CTL family [29]. Subunits of nAChR *α*1, *α*2, *α*5, *α*7, *α*10, *β*1, *β*2, and *γ* and mAChR M2, M3, M4, and M5 were all detected in rat maxilla, and their expression showed significant changes during the expansion, suggesting their involvement in the bone remodeling, but the detail functions and regulation mechanisms still need further investigation.

Besides, an interesting phenomenon was observed that the genes of nonneuronal components were affected by the midpalatal expansion in the same pattern. The expression levels of CarAT, VAChT, AChE, BChE, OCT1, SLC22a4 (Octn1), and nAChR subunits *α*1, *α*2, *α*5, *α*7, *α*10, *β*1, *β*2, and *γ* and mAChR subunits M2, M3, M4, and M5 decreased significantly after 1 day of expansion and then increased slightly after 3 days of expansion, but still lower than the control group. After 7 days of expansion, there was a sharp rise in the expression levels of these genes. The changing pattern of these genes was not coincident with the ACh peak appearing at day 3 after midpalatal expansion, but these cholinergic activities seemed to promote bone mass accrual by complex cellular regulatory networks. some studies [13, 30] showed that acetylcholine system components also had some nonclassical properties in nonneuronal cells such as the association of AChE with adhesion. Blockade of some functional sites of AChE molecule resulted in a concentration-dependent decrease in osteoblastic cell adhesion. These clues make us open our minds to image that maybe some other mechanisms exist in the bone remodeling activities relating to nonneuronal components.

In this work, we investigated the existence of nonneuronal cholinergic system components in rat maxilla and demonstrated the wide presence of most components such as CarAT, VAChT, AChE, BChE, OCT1, SLC22a4 (Octn1), and nAChR subunits *α*1, *α*2, *α*5, *α*7, *α*10, *β*1, *β*2, and *γ* and mAChR subunits M2, M3, M4, and M5. By the rat maxilla expansion model, we also verified the involvement of ACh and the other cholinergic components in the bone remodeling* in vivo *and the results indicated that ACh might play an important role in bone formation. However, there are some deficiencies in our study. Limited by the animal ethics, the number of experimental animals was relatively insufficient though the results were evident. Besides, as a preliminary study, to further understand the detailed functions and mechanisms of ACh and the other nonneuronal cholinergic system components, much more investigations need to be implemented on the basis of this work.

## Supplementary Material

In the Supplementary Material, primer sequences of all the genes tested by Real-Time PCR in this work were provided in Table S1. Statistical analysis of the Real-Time PCR results was provided in Table S2.
