# Supplementary material for: Involvement of the Nonneuronal Cholinergic System in Bone Remodeling in Rat Midpalatal Suture after Rapid Maxillary Expansion
Source: Biomed Res Int. 2016 Jul 10;2016:8106067. doi: 10.1155/2016/8106067 (PMC4958416; doi:10.1155/2016/8106067)
Supplement: Supplementary file 1 — In the Supplementary Material, primer sequences of all the genes tested by Real-Time PCR in this work were provided in Table S1. Statistical analysis of the Real-Time PCR results was provided in Table S2. [file 8106067.f1.pdf]

Supplementary Table 1. Sequences of the primers used for Real-Time PCR.

Gene name	Genbank	Forward	Reverse
ChAT	NC_005115	GCCTGCTGCAACCAGTTCTT	TCGTTGGACGCCATTTTGA
CarAT	NM_001004085	ATTGTCGCTCTTGTGGACC	TCTGTTTGGCCTTCTCTATGTC
VAChT	X80395	GCCACATCGTTCACTCTCTTG	CGGTTCATCAAGCAACACATC
AChE	S50879	CTTCTCCCACACCTGTCC	CTGCTTCCTGGTAGAGCC
BChE	NM_022942	ACCTAACCTTGAACACAGAGAAG	TTCCACTCTTGCTCCCTTTC
CHT1	NC_005108	ATGGAACTTGCTGATAGGATGGA	ATGATTCTCCCGATTGTTCTACAG
OCTN2	NM_019269	CGATCCCAGTGAGTTACAAGAC	GAGAAAGTCCGAAGTAGCCC
OCT1	NM_012697	TGGCCGTAAGCTCTGTCTCT	TCAAGGTATAGCCGGACACC
OCT2	NM_031584	ATGCTGGACCTGTTTCAGTC	AGAAATCGCCATGAGAGCC
OCT3	NM_019230	CAATGGGAAACACCTCTCGT	ATACACCACGGCACTTGTGA
SLC22a4	NM_022270	CTGGGAGTACAGCAAGGA	GAAGGAGCCACAGAGAAC
SLC25a20	NM_053965	AACCCATCAGTCCGCTTAAG	TGGTCCCAGAGTACATAGGTG
SLC25a29	NM_001010958	CCCGAGGAGTAGTAGGACTCAG	CACAATCACACCTGCCACA
SLC22a8	NM_031332	AGCACCAGAGACACCATTG	ACAGTTCTCCAATCACAGGTC
α1	NM_024485	GTCACCCACTTTCCCTTCGA	CAGGTCGGGCTGGTCACTT
α2	NM_133420	CGCTGGTCATCCCACTCAT	GGGAGCGGTGGTGTACATTG
α3	NM_052805	TCCAGTTTGAGGTGTCCATG	CTTGGTAGTCAGAGGGTTTCC
α4	NM_024354.1	CGCATCCCCTCTGAACTCAT	CACTGCACCCTTCCGTCATA
α5	NM_017078	TGGACGCAACCAGCAAACTA	TATGTCCACGAGCCGAATTTC
α7	AY574256	GCTGGTTCCCTTTTGATG	CTCCGTTGGGGATATAGC
α9	RRU12336	CGCGGTGCTGAATGTTACAC	GTACGCATCGTGCCAGGTTT
α10	AF196344	CAGTCTCTCCCCAAAGTG	GAGGTGGGCTTTAGATCC
β1	X74833	TGGTTGTGGACCGTCTTTTTC	ATGACCGGAGGGTCCTCAAG
β2	L31622	TTCCTGCTGCTCATCTCCAA	CGCTGGTGACGATGGAGAA
β3	NM_133597.1	TGGAAACACTCTGCGCTTGA	TCCTGCAGTGGCCGTAAGA
β4	AY574260	TGCTGGCACTCACGTTCTTC	AAGGTGACCAGCACCATGGT
delta	NM_019298	GCTCCCTCAAATTCAGTTCAC	ACTATTTCCCACTCACCGTTC
epsilon	NM_017194	CAATGTTCTGGTCTATGAGGGAG	CTTCAGCATTGTAGGTCTGGG
gamma	NM_019145	CACCTACTTCCCCTTCGATTG	ATTCTCTGTGAAAGCCTCGG
M1	NM_080773	TGGTTTCCTTCGTTCTCTGG	GAGGAACTGGATGTAGCACTG
M2	J03025	CCACTCCAGAGATGACAACT	GGCTACAACGTTCTGCTTT
M3	M16407	GGACTGTGGATGTGGAGAG	CGAGGAGTTGGTGTCAGA
M4	NM_031547	CCCGCCGCACTACTAAGATG	CCTCTTGCCCACCACAAACT
M5	M22926	CAGCTGCTGCTCACAGACTCA	GGGAAGGAACAGGGCATGAT
RANKL	NM_057149	AGCGCTTCTCAGGAGTTCCA	GCCGGGCCACATCGA

OPG	U94330.1	GCTGGCACACGAGTGATGAA	CGGTCTGCAGTTCCTTGCA
β-actin	NM_031144	CTTCAACACCCCAGCCATGT	CAGAGGCATACAGGGACAACA
18S	M11188	GCGGTTCTATTTTGTTGG	AATGCTTTCGCTCTGGTC

Supplementary Table 2. Statistical data from Real-Time PCR results.

Gene	2-ΔΔCt (Mean±SD)				
	**C**	**E1**	**E3**	**E7**	**p-value**
OPG	0.668±0.311	0.900±0.418	0.776±0.381	0.161±0.087	0.187
RANKL	0.651±0.357	11.015±1.302	0.229±0.050	0.183±0.126	0.102
RANKL/OPG	0.928±0.270	8.354±0.624	0.348±0.162	1.034±0.320	0.014*
AChE	0.740±0.233	0.006±0.068	0.065±0.012	14.562±11.347	0.007*
BChE	0.645±0.242	0.066±0.049	0.132±0.206	3.882±1.610	0.000*
CarAT	0.520±0.321	0.197±0.281	0.150±0.218	0.205±0.032	0.218
VAChT	0.702±0224	0.007±0.008	0.034±0.010	11.769±10.938	0.012*
SLC22a4	0.717±0.208	0.060±0.054	0.260±0.263	6.610±5.506	0.009*
OCT1	0.616±0.325	0.006±0.045	0.020±0.021	26.990±17.263	0.009*
OCT3	0.486±0.381	0.019±0.095	0.076±0.115	1.487±0.810	0.025*
SLC25a20	0.497±0.348	0.026±0.017	0.289±0.438	0.227±0.083	0.090
nAChR α1	0.807±0.224	0.005±0.003	0.100±0.098	35.44±7.32	0.011*
nAChR α2	0.620±0.294	0.010±0.0098	0.041±0.022	13.644±16.194	0.003*
nAChR α3	0.415±0.409	0.064±0.070	0.076±0.078	2.917±2.242	0.027*
nAChR α5	0.856±0.124	0.050±0.050	0.223±0.108	24.474±14.315	0.012*
nAChR α7	0.663±0.260	0.061±0.027	0.197±0.146	32.164±33.129	0.014*
nAChR α10	0.459±0.370	0.066±0.094	0.029±0.024	15.363±15.908	0.012*
nAChR β1	0.617±0.287	0.046±0.040	0.162±0.164	24.120±19.109	0.009*
nAChR β2	0.702±0.335	0.023±0.012	0.258±0.198	11.771±17.588	0.013*
nAChR β4	0.422±0.392	0.993±0.917	4.999±6.654	5.325±7.679	0.432
nAChR γ	0.692±0318	0.006±0.0075	0.0003±0.0005	1.183±0.080	0.011*
mAChR 1	0.631±0.332	0.056±0.092	0.426±0.472	14.472±16.859	0.021*
mAChR 2	0.438±0.354	0.058±0.084	0.039±0.033	7.000±5.898	0.007*
mAChR 3	0.779±0.165	0.051±0.068	0.097±0.088	22.186±22.227	0.009*
mAChR 4	0.773±0.245	0.029±0.045	0.103±0.149	16.180±17.270	0.009*
mAChR 5	0.576±0.337	0.022±0.010	0.143±0.078	19.734±17.805	0.009*

* statistically significant
